# The Treatment With the SGLT2 Inhibitor Empagliflozin Modifies the Hepatic Metabolome of Male Zucker Diabetic Fatty Rats Towards a Protective Profile

**DOI:** 10.3389/fphar.2022.827033

**Published:** 2022-02-02

**Authors:** Alana Aragón-Herrera, Manuel Otero-Santiago, Laura Anido-Varela, Sandra Moraña-Fernández, Manuel Campos-Toimil, Tomás García-Caballero, Luis Barral, Estefanía Tarazón, Esther Roselló-Lletí, Manuel Portolés, Oreste Gualillo, Isabel Moscoso, Ricardo Lage, José Ramón González-Juanatey, Sandra Feijóo-Bandín, Francisca Lago

**Affiliations:** ^1^ Cellular and Molecular Cardiology Research Unit, Institute of Biomedical Research and Xerencia de Xestión Integrada de Santiago (XXIS/SERGAS), Santiago de Compostela, Spain; ^2^ Centro de Investigación Biomédica en Red de Enfermedades Cardiovasculares (CIBERCV), Institute of Health Carlos III, Madrid, Spain; ^3^ Group of Pharmacology of Chronic Diseases (CD Pharma), Department of Pharmacology, Pharmacy and Pharmaceutical Technology, University of Santiago de Compostela, Santiago de Compostela, Spain; ^4^ Department of Morphological Sciences, University of Santiago de Compostela and Xerencia de Xestión Integrada de Santiago (XXIS/SERGAS), Santiago de Compostela, Spain; ^5^ Group of Polymers, Department of Physics and Earth Sciences, University of La Coruña, La Coruña, Spain; ^6^ Cardiocirculatory Unit, Health Research Institute of La Fe University Hospital, Valencia, Spain; ^7^ Laboratory of Neuroendocrine Interactions in Rheumatology and Inflammatory Diseases, Institute of Biomedical Research and Xerencia de Xestión Integrada de Santiago (XXIS/SERGAS), Santiago de Compostela, Spain; ^8^ Cardiology Group, Center for Research in Molecular Medicine and Chronic Diseases (CIMUS) and Institute of Biomedical Research of Santiago de Compostela (IDIS-SERGAS), Universidade de Santiago de Compostela, Santiago de Compostela, Spain

**Keywords:** empagliflozin, diabetes, liver, metabolome, inflammation

## Abstract

The EMPA-REG OUTCOME (Empagliflozin, Cardiovascular Outcome Event Trial in patients with Type 2 Diabetes Mellitus (T2DM)) trial evidenced the potential of sodium-glucose cotransporter 2 (SGLT2) inhibitors for the treatment of patients with diabetes and cardiovascular disease. Recent evidences have shown the benefits of the SGLT2 inhibitor empagliflozin on improving liver steatosis and fibrosis in patients with T2DM. Metabolomic studies have been shown to be very useful to improve the understanding of liver pathophysiology during the development and progression of metabolic hepatic diseases, and because the effects of empagliflozin and of other SGLT2 inhibitors on the complete metabolic profile of the liver has never been analysed before, we decided to study the impact on the liver of male Zucker diabetic fatty (ZDF) rats of a treatment for 6 weeks with empagliflozin using an untargeted metabolomics approach, with the purpose to help to clarify the benefits of the use of empagliflozin at hepatic level. We found that empagliflozin is able to change the hepatic lipidome towards a protective profile, through an increase of monounsaturated and polyunsaturated glycerides, phosphatidylcholines, phosphatidylethanolamines, lysophosphatidylinositols and lysophosphatidylcholines. Empagliflozin also induces a decrease in the levels of the markers of inflammation IL-6, chemerin and chemerin receptor in the liver. Our results provide new evidences regarding the molecular pathways through which empagliflozin could exert hepatoprotector beneficial effects in T2DM.

## Introduction

Type 2 diabetes mellitus (T2DM) is highly connected to metabolic liver disease, that is a chronic condition that implies hepatic fat accumulation associated to underpinning metabolic dysregulation (Lim et al., 2021) and low-grade inflammation (Furman et al., 2019; Watt et al., 2019; Lim et al., 2021). This complex and bidirectional association raises the possibility of common mechanisms of disease treatment (Vincent et al., 2020; Gastaldelli et al., 2021). With the increasing interest in the non-glycaemic effects of the pharmacological treatments used for diabetes control, several recent clinical trials and translational studies have explored the hepatic effects linked to the use of anti-diabetic drugs such as the analogues of glucagon-like peptide one or the sodium glucose cotransporter-2 (SGLT2) inhibitors (Ranjbar et al., 2019; Cusi, 2020; Kang et al., 2020; Vincent et al., 2020). The SGLT2 inhibitors are new oral anti-diabetic agents that promote renal glucose excretion in an insulin-independent mechanism, being well established in the treatment of T2DM (Frampton, 2018). One of the four SGLT2 inhibitors approved by the US Food and Drug Administration (FDA) is empagliflozin that, both in patients with or without T2DM, has demonstrated to have additional renoprotective and cardioprotective effects that are independent of the glycaemic control (Zinman et al., 2015; Täger et al., 2020). Although the pathways through which SGLT2 inhibitors exert those beneficial effects are still unknown, it has been widely suggested that energy metabolism regulation and reduction of inflammation could be two of the mechanisms more tightly implicated (Bonnet and Scheen, 2018; Flores et al., 2018; Verma and McMurray, 2018). Particularly, empagliflozin treatment has been shown to be able to reduce fat hepatic content in T2DM patients with fatty liver disease (Kuchay et al., 2018) and in animal models of diabetes-associated steatohepatitis (Jojima et al., 2016; Petito-da-Silva et al., 2019; Xu et al., 2019).

We have demonstrated earlier that, in Zucker diabetic fatty (ZDF) rats, the treatment with empagliflozin modifies the cardiac metabolome, greatly reducing the content of lipid species associated with cardiac lipotoxicity (Aragón-Herrera et al., 2019). In the present work, we aimed to broaden our previous work to study the impact of this therapy on liver metabolome, as well as on the levels of inflammation in hepatic tissue.

## Methods

All reagents and materials are from Sigma-Aldrich (United States) except where stated otherwise.

### Ethical Statement

The study was performed in accordance with the ARRIVE guidelines (Animals in Research: Reporting *In Vivo* Experiments) (Kilkenny et al., 2010) and the European Union Directive 2010/63. The protocol used was approved by the Galician Clinical Research Ethics Committee (2007/304) (protocol number 15005/2015/003).

### Animal Care

Zucker Diabetic Fatty (ZDF) rats (ZDF-Lepr^fa/fa^) are a model of early stage T2DM with high insulin and glucose intolerance in liver and skeletal muscle (Shiota and Printz, 2012). Male ZDF-Lepr^fa/fa^ rats with a body weight ranging 200–250 g were obtained from Charles River Laboratories (United States). In order to induce T2DM, animals were fed *ad libitum* with the diabetogenic diet Formulab 5008 (LabDiet, United States) according to the supplier instructions, starting at 7-weeks of age. Rats were housed at the Animal House of the CiMUS (Centro Singular de Investigación en Medicina Molecular y Enfermedades Crónicas, Santiago de Compostela, Spain) under controlled room temperature (22 ± 2°C), relative humidity (40–5%) and illumination (12-h light/12-h dark cycle), with free access to chow and water.

### Empagliflozin *In Vivo* Treatment

Once ZDF rats reached fasting glucose levels of 350.75 ± 18.59 mg/dl (12-weeks old), they were divided in 2 random groups: control (vehicle, mineral drinking water) (*n* = 15) and treated (30 mg/kg/d empagliflozin in the drinking water (Boehringer Ingelheim Pharma GmbH&Co. KG, DEU)) (*n* = 15) (Steven et al., 2017; Aragón-Herrera et al., 2019). After 6 weeks of treatment, animals were euthanized and the livers (which were collected during 9:00 to 11:00 h) were quickly frozen in liquid nitrogen and stored at –80 °C until being shipped in dried ice to One Way Liver Metabolomics (OWL metabolomics S.L., ESP) for metabolomic analysis. Livers employed in the present study were harvested from the animals used in our previous work (Aragón-Herrera et al., 2019).

### Blood Glucose Measurement

Animal nourishments were retired at 20:00 h, and after 12 h of fasting, a blood sample from the tail vein was collected and measured using the glucometer GlucoDr auto™ (All Medicus Co. Ltd., KOR) once a week.

### Quantitative Real-Time Polymerase Chain Reaction

The hepatic gene expression of interleukin-6 (IL-6) (RefSeq NM_012,589.2), retinoic acid receptor responder 2 or chemerin (RARRES2) (RefSeq NM_001013427.1), chemerin chemokine-like receptor 1 (CMKLR1) (RefSeq NM_022,218.2) and 18S ribosomal RNA (18S) (RefSeq NR_046,237.1) as a housekeeping gene were determined by RT-qPCR. The NucleoSpin RNA Mini kit for RNA purification (Macherey-Nagel, DEU) was used to extract total RNA. Subsequently, the extracted RNA was measured in a NanoDrop 1000 spectrophotometer (Thermo Fisher Scientific, United States) to quantify and evaluate its purity. cDNA was synthetized performing a reverse transcription from 1 µg of RNA, using the Transcriptor First Strand cDNA Synthesis Kit (Thermo Fisher Scientific, United States) according to manufacturer’s instructions. RT-qPCR was performed using RT^2^ SYBR Green qPCR Mastermix and specific primers provided by Thermo Scientific using a Stratagene MX3000p thermocycler according to manufacturer’s instructions (Agilent Technologies, United States).

### UHPLC-MS Metabolomics Determination

Liver metabolomics analysis (*n* = 10) was carried out by OWL Metabolomics S.L. (ESP) as previously described (Barr et al., 2012; Aragón-Herrera et al., 2019). Briefly, two separate ultra-high performance liquid chromatography (UHPLC)−time-of-flight (TOF)-MS-based platforms for the analysis of methanol and chloroform/methanol extracts were linked to a UHPLC-single quadrupole-MS based platform to analyze amino acids and with a methanol/water extract platform that covered polar metabolites. Lipid nomenclature and classification follows the LIPID MAPS convention (www.lipidmaps.org).

### Statistical Analysis

All data are expressed as mean ± standard error of the mean (SEM), and significance was defined as *p* < 0.05. Comparisons between groups were analyzed with the non-parametric test Wilcoxon-signed rank test using the software GraphPad Prism 8 (GraphPad Software Inc., USA). For gene expression analysis, the fold change *vs.* control in the expression of target genes was calculated using the 2^−ΔΔ*C*t^ method.

For metabolomics analysis, data were pre-processed using the TargetLynx application manager for MassLynx 4.1 (Waters Corp., USA). Intrabatch and interbatch normalization followed the protocol detailed in Martínez-Arranz et al., 2015. Statistical comparisons between groups were analyzed using the Student’s t-test and the software package R v.3.1.1 (R Development Core Team, 2011; AUT; https://cran.r-project.org/).

## Results

### Empagliflozin Effect on Glucose Lowering, Food Intake and Body Composition in Diabetic ZDF Rats

Empagliflozin treatment of diabetic ZDF rats starting at the onset of T2DM significantly decreased fasting glucose levels compared to control (115.9 ± 7.50 mg/dl and 400.9 ± 15.22 mg/dl respectively; *p* < 0.0001, *n* = 15) (Aragón-Herrera et al., 2019).

Before starting the treatment, the rats in the control group weighted 251.1 ± 12.67 g and the empagliflozin group weighted 248.6 ± 13.34 g (p: 0.662, *n* = 15). After 6 weeks of treatment, we observed a slight increase in body weight in the empagliflozin group compared to control (415.8 ± 6.46 g and 387.2 ± 7.71 g respectively, p: 0.0076, *n* = 15), without affecting food intake. As well, fat distribution and muscle volume were unaltered after empagliflozin treatment compared to control (Aragón-Herrera et al., 2019).

Plasma levels of triglycerides (control: 760.5 ± 66.83 mg/dl; empagliflozin: 752.7 ± 52.49 mg/dl; p: 0.808, *n* = 15), cholesterol (221.1 ± 6.96 mg/dl and 233.7 ± 7.70 mg/dl respectively; p: 0.268, *n* = 15), LDL (26.94 ± 1.43 mg/dl and 36.63 ± 7.80 mg/dl; p: 0.395, *n* = 15), HDL (88.31 ± 2.86 mg/dl and 87.44 ± 2.94 mg/dl; p: 0.689, *n* = 15), and the hepatic enzymes alanine transaminase (ALT) (160.7 ± 16.99 UI/L and 133.3 ± 14.81 UI/L; p: 0.243, *n* = 10) and gamma-glutamyl transferase (GGT) (4.75 ± 1.11 UI/L and 2.60 ± 0.46 UI/L; p: 0.304, *n* = 15) were unaltered after empagliflozin treatment (Aragón-Herrera et al., 2019).

We also determined if empagliflozin could affect liver weight by normalizing liver weight by whole body weight. We observed that empagliflozin did not affect liver weight compared to control rats (49.14 ± 0.99 mg/g and 48.55 ± 0.91 mg/g respectively; p: 0.555, *n* = 5).

### Empagliflozin Effect on the Hepatic Metabolome in Diabetic ZDF Rats

**FIGURE 1:**
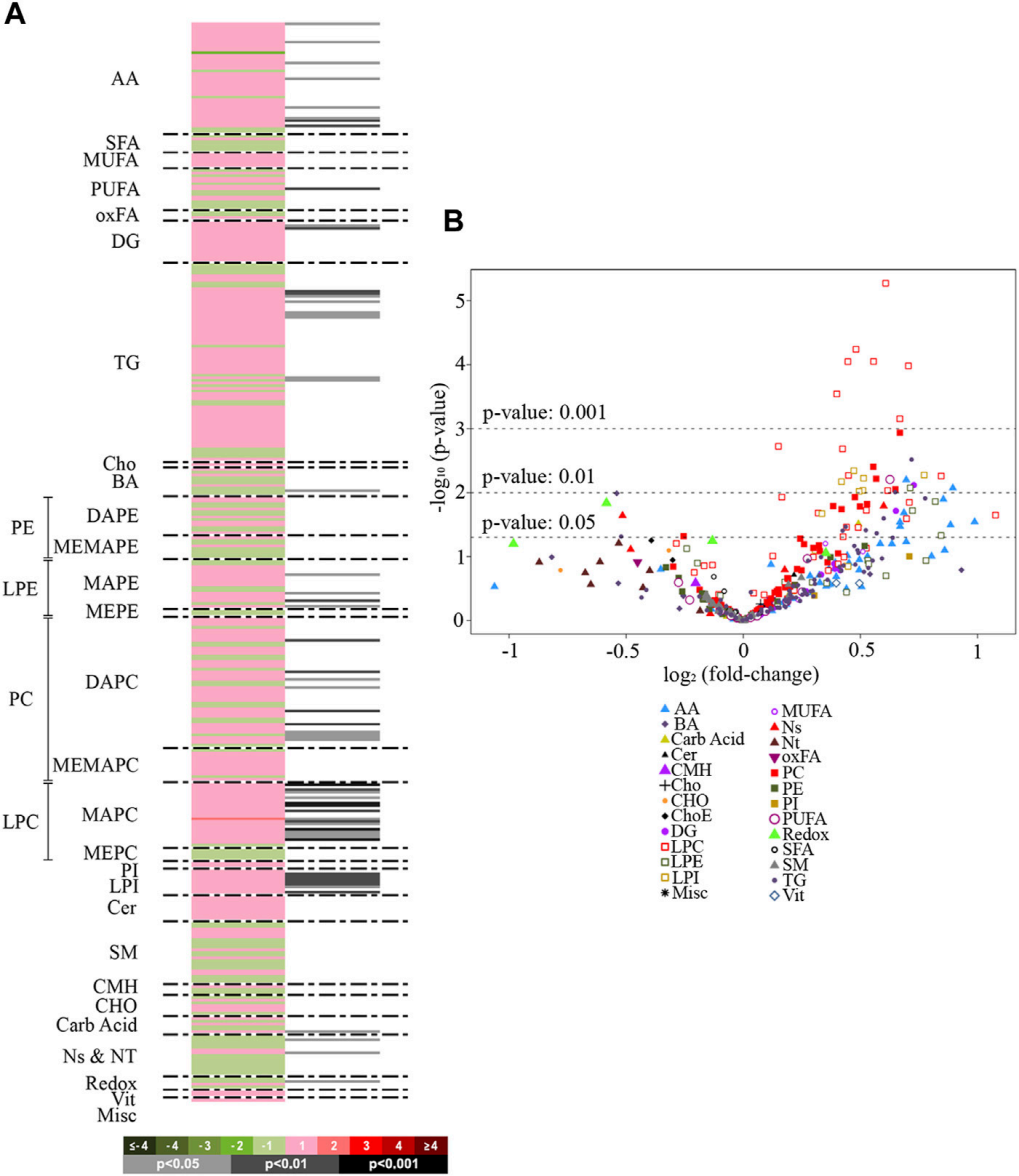
Heat map and volcano plot. **(A)**: Heatmap representing individual metabolic features from rat livers treated with empagliflozin *vs.* control. Green sections of the heatmap denote reduced metabolites (negative log_2_ fold-changes) and red sections denote metabolites increased (positive log_2_ fold-changes). Grey/black bars indicate significant *p*-values of the unpaired Student’s t-test (light grey, *p* < 0.05; dark grey, *p* < 0.01; black, *p* < 0.001). Metabolites are ordered according to their carbon number and unsaturation degree of their acyl chains. **(B)**: Volcano plot [-log_10_(*p*-value) *vs.* log_2_(fold-change)] for the comparison empagliflozin *vs.* control. The graph shows the most significant (*p* < 0.001 and *p* < 0.01) metabolites considered individually. AA, amino acids; SFA, saturated fatty acids; MUFA, mono-unsaturated fatty acids; PUFA, polyunsaturated fatty acids; oxFA, oxidized fatty acids; DG, diacylglycerides; TG, triacylglycerides; Cho, cholesterol; BA, bile acids; PE, phosphatidylethanolamines; DAPE, diacylglycerophosphatidylethanolamines; MEMAPE, 1-acyl,2-etheracylglycerophosphatidylethanolamines; LPE, lysophosphatidylethanolamines; MAPE, monoacylglycerophosphatidylethanolamines; MEPE, monoetherglycerophosphatidylethanolamines; PC, phosphatidylcholines; DAPC, diacylglycerophosphatidylcholines; MEMAPC, 1-acyl,2-etherglyceropho- sphatidylcholines; LPC, lysophosphatidylcholines; MAPC, monoacylglyceropho-sphatidylcholines; MEPC, monoetherglycerophosphatidylcholines; PI, phospatidylinositols; LPI, lysophosphatidylinositols; LPG, cardiolipins; Cer, ceramides; SM, sphingomyelin; CMH: monohexosylceramides, Carb Acid., carboxylic acids; Ns and Nt, nucleosides and nucleotides; Redox, molecules participating in redox homeostasis; Vit., vitamins; Misc., miscellany.

**FIGURE 2:**
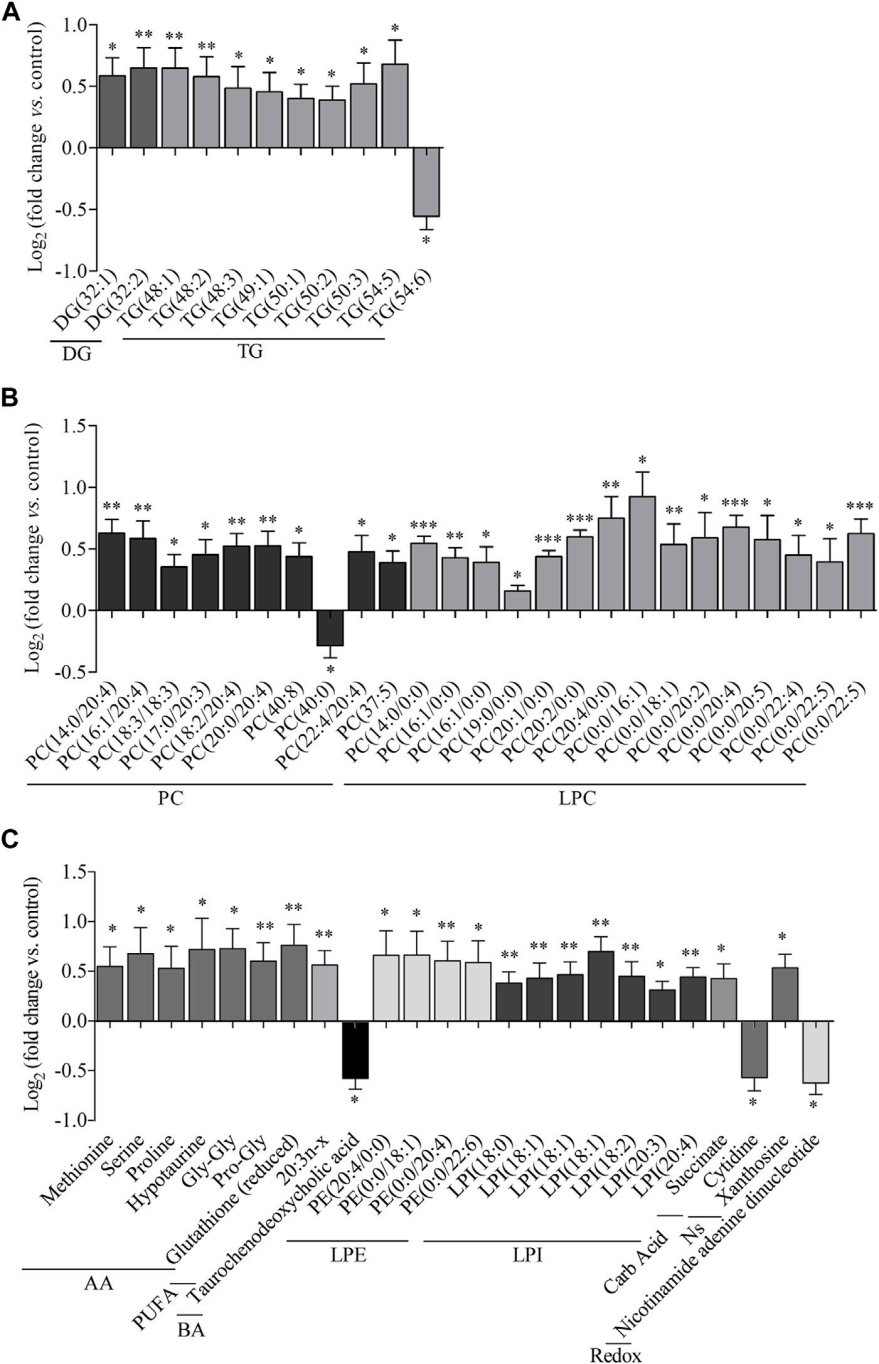
Metabolic features altered in the liver by empagliflozin treatment. Metabolomic analysis of liver tissue from rats treated with empagliflozin *vs* control. Relative log_2_(fold-change) of glycerides **(A)**, phosphatidylcholines and lysophosphatidylcholines **(B)**, amino acids **(C)**, polyunsaturated fatty acids **(C)**, bile acids **(C)**, lysophosphatidylethanolamines **(C)**, lysophosphatidylinositols **(C)**, carboxylic acids **(C)** and redox molecules **(C)**. DG, diacylglycerides; TG, triacylglycerides; PC, phosphatidylcholines; LPC, lysophosphatidylcholines; AA, amino acids; PUFA, polyunsaturated fatty acids; BA, bile acids; LPE, lysophosphatidylethanolamines; LPI, lysophosphatidylinositols; Carb Acid., carboxylic acids; Redox, molecules participating in redox homeostasis. *p < 0.05, **p < 0.01, ***p < 0.001.

A total of 400 metabolites were detected in the analyzed liver samples. Among these, 16 metabolites were found to be outliers and were excluded from the statistical analysis. The heatmap in Figure 1A displays the log2 (fold-change) of the 384 metabolites included in the study, showing that livers from rats treated with empagliflozin had higher levels of several amino acids (7), diglycerides (DG) (2), triglycerides (TG) (9), monoacylglycerophosphoethanolamines (4), diacylglycerophosphocholines (8), monoacylglycerophosphocholines (15) and lysophosphatidylinositols (LPI) (8). A volcano plot [-log10(*p*-value) *vs.* log2(fold-change)] was also generated for comparison between empagliflozin *vs*. control (Figure 1B). This volcano emphasizes the most significant altered metabolites considered individually for this comparison and complements the heatmap. Most of the altered metabolites were increased in treated rats compared to controls. Remarkably, all significantly increased glycerolipids (DG and TG) were monounsaturated or polyunsaturated species, while changes in saturated features were not found (Figure 1A and Figure 2). Changes in phosphatidylcholines (PC) were also relevant, reaching *p*-values < 0.001 for some of them. Only diacylglycerophosphocholines and monoacylglycerophosphocholines were significantly augmented (Figures 1A, 2). In contrast, ether-linked species were not altered with the treatment (1-ether, 2-acylglycerophosphocholines or 1-Monoetherglycerophosphocholine). We did not find any difference between control and empagliflozin-treated rats regarding total cholesterol (1.1 ± 0.6 relative intensity in arbitrary units (a.u.) and 1.05 ± 0.07 (a.u.) respectively; p: 0.554, *n* = 10) and total triglycerides (63.70 ± 5.38 (a.u.) and 54.01 ± 7.81 (a.u.); p: 0.320, *n* = 10) content in the liver.

**FIGURE 3:**
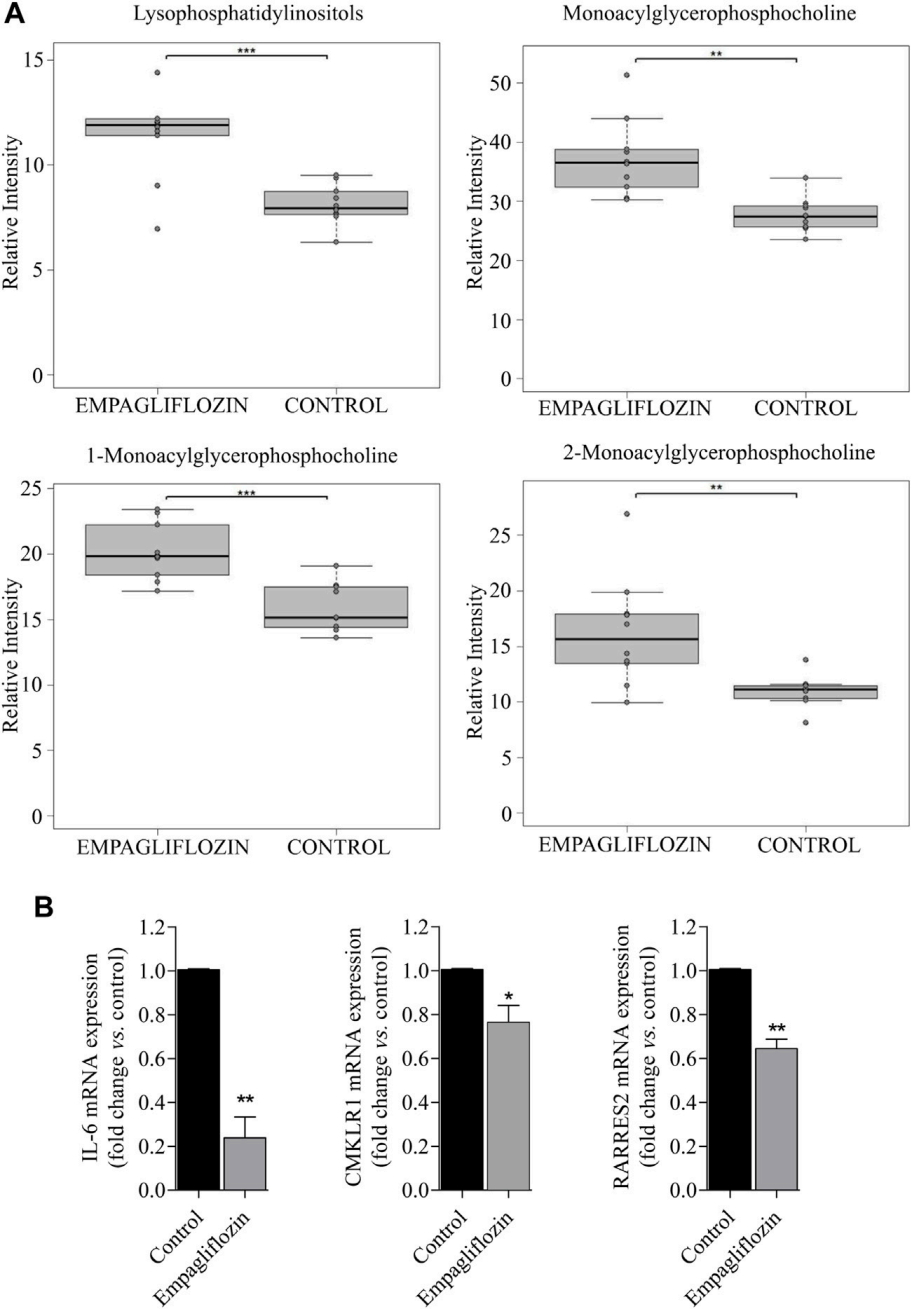
Metabolic classes and pro-inflammatory markers altered in the liver by empagliflozin treatment. **(A)**: Boxplots of lysophospholipids for the comparison empagliflozin *vs.* control. **(B)**: Statistical analysis of RT-PCR for RARRES2 (chemerin), chemerin receptor (CMLKR1) and IL-6 in the liver of diabetic ZDF rats treated with empagliflozin *vs.* control. a.u: arbitrary units. *p < 0.05, **p < 0.01, ***p < 0.001.

Regarding other metabolic species, increased levels of amino acids, a fatty acid, monoacylglycerophosphoethanolamines, succinate and xanthosine were found in livers from rats treated with empagliflozin (Figures 1A, 2). Only 3 metabolites were significantly decreased in this group (taurochenodeoxycholic acid, cytidine and nicotinamide adenine dinucleotide) compared to controls (Figure 2). In addition to the individual metabolites, we studied the changes in metabolic classes between empagliflozin-treated and control rats, calculated as the sum of the normalized areas of all the metabolites with the same chemical characteristics. Significantly altered metabolic classes for the comparison empagliflozin *vs.* control are summarized in Table 1, being the most relevant glycerophospholipids, especially monoacyl-species, and aromatic amino acids. Some of the biggest changes in the metabolic classes are represented in the boxplots shown in Figure 3A.

**TABLE 1:** Significantly altered metabolic classes and enzyme ratios in liver from empagliflozin-treated rats compared to control rats. TG, triacylglycerides; FA, fatty acids; PUFA, polyunsaturated fatty acids; MUFA, mono-unsaturated fatty acids; BCAAs, branched chain amino acids; ChoE, cholesteryl esters; Cho, cholesterol; PC, phosphatidylcholines; PE, phosphatidylethanolamines; DHA, docosahexaenoic acid; SM, sphingomyelin.

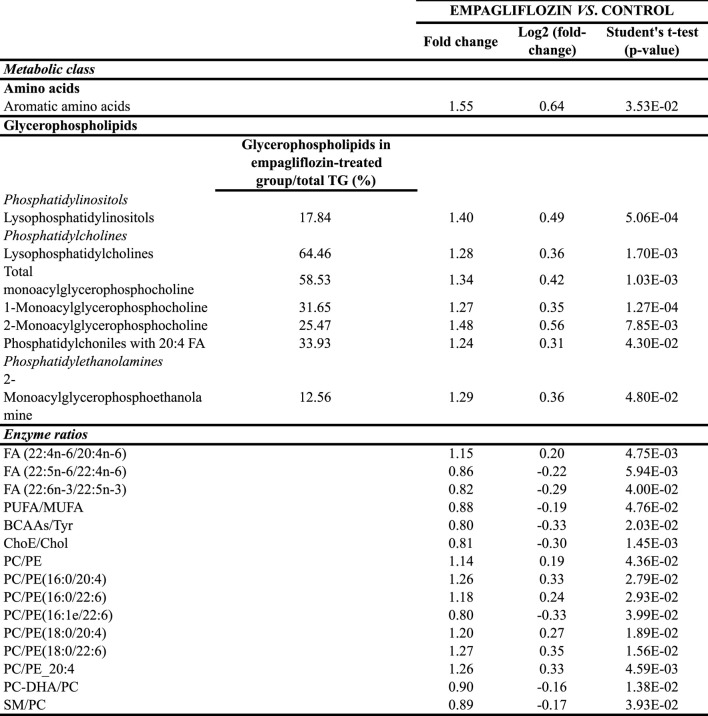

Similarly, in order to know the potential enzyme activities related to hepatic lipid metabolism, we calculated several enzyme ratios (Table 1). We found the ratios (PC)/phosphatidylethanolamine (PE) PC/PE(16:0/20:4), PC/PE(16:0/22:6), PC/PE(18:0/20:4) and PC/PE(18:0/22:6) increased after empagliflozin treatment compared to controls, what may suggest that methylation by phosphatidylethanolamine N-methyltransferase (PEMT) could be increased in the livers of rats treated with empagliflozin (Table 1).

### Empagliflozin Effect on Pro-inflammatory Markers in the Liver

Empagliflozin treatment induces a reduction on hepatic gene expression of the pro-inflammatory markers chemerin, chemerin receptor and IL-6 (Figure 3B) in diabetic ZDF rats compared to untreated controls.

## Discussion

The most relevant result in our present work is the demonstration that the treatment with empagliflozin is able to modify the hepatic metabolome of male ZDF rats into a more favorable profile, and that this SGLT2 inhibitor is also able to decrease the gene expression of pro-inflammatory markers in the liver of those animals.

The ZDF rat is a model of T2DM well established, and it is also used as a model for the study of metabolic fatty liver disease (Shiota and Printz, 2012; Kucera, 2014). In our work, we observed an increase in hepatic DG and TG species containing MUFAs and PUFAs after empagliflozin treatment of ZDF diabetic rat, whereas no changes in saturated species were found. Although hepatic glycerides can be raised in metabolic liver disease, increasing data show that the total quantity of TG accumulated in the hepatic cells is not the principal cause of lipotoxicity, and that are specific classes of lipids (mainly saturated forms) those that act as damaging agents (Svegliati-Baroni et al., 2019). Moreover, it has also been reported that usually MUFAs and PUFAs species are decreased in metabolic liver disease (Kotronen et al., 2009; Barr et al., 2010; Chitraju et al., 2012). The acyl chain composition of DG and TG seems to be critical to determine the effects derived from their increase at hepatic level and, indeed, it has been recently demonstrated that glycerides that contain fatty acids that are longer and more unsaturated accumulate in larger amounts in the liver during adaptive thermogenesis, a process known to be linked to an improved metabolic profile (Stanford et al., 2013; Bartelt et al., 2017; Grefhorst et al., 2018; Pernes et al., 2021). Moreover, it has been also proposed that an enrichment of MUFAs and PUFAs in hepatic non-toxic-TG pools can prevent the lipotoxicity and the endoplasmic reticulum (ER) stress at hepatic level (Fuchs et al., 2012). In concordance with this, Nasiri-Ansari et al. (2021) have recently demonstrated that empagliflozin is able to reduce the endoplasmic reticulum stress in the liver of ApoE(−/−) mice. To note, and in consonance with our data, Ueta et al. (2014) and O’Brien et al. (2017) have demonstrated that the treatment with SGLT2 inhibitors was able to reduce the TG content of skeletal muscle, but not the hepatic TG content of ZDF rats; while Perakakis et al. (2021) recently reported an increase of the hepatic concentrations of TG in empagliflozin-treated non-diabetic mice with advanced steatohepatitis. The increase in hepatic DG and TG species containing MUFAs and PUFAs after empagliflozin treatment of ZDF diabetic rats might involve changes in the hepatic expression and/or activity of relevant enzymes in MUFA and PUFA biosynthesis, the Δ9, Δ5-and Δ6-fatty acid desaturases (Nakamura and Nara, 2004), whose levels and activity have been found altered in hepatic pathological conditions and in animal models of hepatic disease, and whose deficiency has been associated to liver metabolic diseases (Araya et al., 2010; Chiappini et al., 2017; Gromovsky et al., 2018; Palladini et al., 2019).

In our study, we observed that empagliflozin increases PC and PE hepatic content. Phospholipids play a pivotal role in regulating physiological functions and maintaining cellular membrane structures, integrity and fluidity, being PC and PE the most abundant at hepatocellular level (Cano and Alonso, 2014). Alterations in phospholipid composition can result in cell membranes damage, what has been strongly involved in the aetiology of chronic liver diseases (Hall et al., 2017; Lüchtenborg et al., 2020). In agreement with this, a reduction in the content of PC in the liver has been associated with the development of hepatic steatosis (Niebergall et al., 2011). We also demonstrate here that empagliflozin is able to increase the PC/PE ratio in ZDF rats compared to untreated controls. Anomalous high or low PC/PE ratios in different tissues can affect energy metabolism and have been linked to metabolic disease progression (van der Veen et al., 2017; van der Veen et al., 2019). In the liver, the 70% of PC is generated via the cytidine diphosphate–choline pathway, while the enzyme PEMT accounts for ∼30% of hepatic PC biosynthesis through sequential methylation reactions of PE, and is critical for maintaining the phospholipid balance necessary for the preservation of a healthy liver (Niebergall et al., 2011; Wan et al., 2019). A reduction of the hepatic molar ratio PC/PE (considered a key regulator of cell membrane integrity whose alteration contributes to the development of metabolic hepatic disease) has been found in patients with fatty liver, and it has been associated with a decline in the activity of PEMT in some *in vivo* and *in vitro* conditions (Arendt et al., 2013; Wan et al., 2019; Lüchtenborg et al., 2020; Peng et al., 2021); but several other factors can modulate the ratio PC/PE at hepatic level: for example, it has been reported that the exposure to high levels of free fatty acids lowers hepatocyte PC/PE ratio mainly by increasing the cellular PE level, that was attributable to enhanced production via the cytidine diphosphate-ethanolamine pathway and less consumption *via* the PEMT pathway, with the latter pathway also producing less PC (Peng et al., 2021).

In our work, we found that empagliflozin treatment increases the hepatic content of 3 unsaturated and 3 polyunsaturated LPI contents (included arachidonic acid-containing LPI). To note, a reduction of arachidonic acid-containing species has been recently tightly associated to the pathogenesis of hepatic fibrosis and liver damage (Bianco et al., 2021; Thangapandi et al., 2021). This impairment in hepatic LPI metabolism would involve a reduction in the activity of a LPI acyltransferase (LPIAT1/MBOAT7, that belongs to the membrane-bound O-acyltransferase (MBOAT) family) implicated in the remodelling of phospholipid acyl-chain in the Land’s cycle, incorporating arachidonic acid and different PUFAs within LPI and several more lysophospholipids; thus, it is a fine-tune regulator of the amount of free arachidonic acid, that is a potent trigger for hepatic inflammation and fibrosis (Bianco et al., 2021; Thangapandi et al., 2021).

In this work, we show that empagliflozin treatment increases liver LPC levels in diabetic ZDF rats compared to untreated controls. Interestingly, elevated plasma levels of LPC in patients with non-alcoholic fatty liver have been associated with anti-inflammatory and glucose-lowering effects, and with metabolically benign disease (Lehmann et al., 2013), while decreased levels of LPC have been found in plasma of obese (Heimerl et al., 2014) and insulin-resistant (Rauschert et al., 2016) patients when compared to control individuals. Moreover, in overweight and obese individuals, insulin sensitivity in the muscle is linked to more elevated plasma LPC concentrations (van der Kolk et al., 2019). Since low LPC levels appear to be clearly a marker of metabolic liver disease-connected insulin resistance, our results may reveal novel significant beneficial mechanisms of action of empagliflozin in the liver. As also reported for LPI, it has been demonstrated that LPC are remodeled through the Land’s cycle, whose reacylation step is catalyzed in the liver by LPC acyltransferase 3 (LPCAT3), that belongs to the membrane-bound O-acyltransferase (MBOAT) family, and whose hepatic gene expression is regulated by peroxisome proliferator-activated receptor alpha (PPARα, nuclear receptor that regulates fatty acid oxidation and target genes involved in lipoprotein metabolism) agonists (Zhao et al., 2008). To note, it has been demonstrated that the treatment with empagliflozin is able to increase the hepatic expression of PPARα in high-fat-fed mice (Petito-da-Silva et al., 2019). Our study provides tools for future investigations on how LPI and LPC remodeling may potentially be involved in the hepatic effects of the treatment with empagliflozin in diabetes.

Regarding other metabolic features altered in the liver of ZDF rats by the treatment with empagliflozin, we would like to highlight the decrease in the hepatic content of taurochenodeoxycholic acid, a bile acid whose levels have been positively associated with liver injury (Ma et al., 2019) and cirrhosis in humans (Yang et al., 2019).

Inflammation is a common feature in metabolic disorders, including fatty liver disease (Ismaiel and Dumitraşcu, 2019; Lee and Friedman, 2021). The inflammation of adipose tissue and the insulin resistance drive to an overload of fatty acids and glucose in the liver that causes hepatic steatosis, ER stress and activation of the unfolded protein response, generating the activation of the inflammasome and the cell death (Lee and Friedman, 2021). Hepatocyte damage signals result in the activation of hepatic stellate and inflammatory cells that assemble hepatic fibrogenesis (Lee and Friedman, 2021). In our work we could confirm that empagliflozin induced in ZDF rats a drop in the hepatic gene expression of the inflammatory marker IL-6, what had been previously reported in mice models of non-alcoholic fatty liver disease with diabetes after treatment with this drug (Jojima et al., 2016; Nasiri-Ansari et al., 2021), Our results also show, for the first time, that empagliflozin is able to induce a significant decline in the hepatic gene expression both of the adipokine chemerin and of chemerin receptor. Chemerin is a pro-inflammatory adipokine produced by the white adipose tissue and by several other tissues, including the liver, that has been linked to obesity, insulin resistance, inflammation, and fatty liver disease (Su et al., 2021). Over the past decade chemerin, that was initially identified as a potent chemoattractant in different immune cells with pivotal roles in both innate and adaptive immunity, has increasingly emerged as a biomarker characterizing inflammatory and metabolic phenotypes, and linking low-grade inflammation with metabolic disorders (Helfer and Wu, 2018; Eichelmann et al., 2019; Koelman et al., 2021), having been proposed as an early marker of chronic subclinical inflammation (Koelman et al., 2021). In accordance to this, chemerin has been strongly associated with the pathogenesis and/or progression of hepatic metabolic dysfunction (Bekaert et al., 2016; Jacenik and Fichna, 2020; Léniz et al., 2022).

To note, we have previously described a robust SGLT2 hepatic gene expression in both control and empagliflozin-treated ZDF rats, which is lower but comparable with its expression levels in kidney (Aragón-Herrera et al., 2019). According to this, the metabolic effects of empagliflozin reported in our work might be induced by direct actions at hepatic level of this SGLT2 inhibitor. However, it cannot be ruled out that those effects could derive from the whole-body extensive metabolic changes induced by the treatment with SGLT2 inhibitors in conditions of obesity and diabetes.

In conclusion, the results of this work evidence that the treatment with empagliflozin is able to modify the hepatic metabolome of ZDF rats towards a protective profile, while diminishing the expression of pro-inflammatory molecules tightly involved in the physiopathology of liver metabolic disease. Since SGLT2 inhibitors seem to be an attractive therapeutic opportunity for metabolic liver disease management, these findings come in support of a beneficial effect for empagliflozin in the regulation of hepatic metabolism in a context of obesity and diabetes.

## Data Availability

The original contributions presented in the study are included in the article, further inquiries can be directed to the corresponding author.
